# Detoxification Impacts of Ascorbic Acid and Clay on Laying Japanese Quail Fed Diets Polluted by Various Levels of Cadmium

**DOI:** 10.3390/ani10030372

**Published:** 2020-02-25

**Authors:** Diaa E. Abou-Kassem, Mohamed E. Abd El-Hack, Ayman E. Taha, Jamaan S. Ajarem, Saleh N. Maodaa, Ahmed A. Allam

**Affiliations:** 1 Animal and Poultry Production Department, Faculty of Technology and Development, Zagazig University, Zagazig 44511, Egypt; drdiaaaboukassem_19@yahoo.com; 2 Poultry Department, Faculty of Agriculture, Zagazig University, Zagazig 44511, Egypt; 3 Department of Animal Husbandry and Animal Wealth Development, Faculty of Veterinary Medicine, Alexandria University, Edfina 22758, Egypt; 4 Department of Zoology, College of Science, King Saud University, P.O. Box 2455, Riyadh 11451, Saudi Arabia; jajarem@KSU.EDU.SA (J.S.A.); maodaa_28@yahoo.com (S.N.M.); 5 Department of Zoology, Faculty of Science, Beni-suef University, Beni-suef 65211, Egypt; allam1081981@yahoo.com

**Keywords:** cadmium, ascorbic acid, clay, performance, blood biochemical parameters

## Abstract

**Simple Summary:**

The present study aimed to evaluate the impacts of ascorbic acid and clay supplementation on laying Japanese quail fed diets polluted by various levels of cadmium (Cd). Results revealed that consuming polluted diets with Cd causes harmful impacts on the productive performance of laying Japanese quail. The supplementation of ascorbic acid or natural clay to layer diets had beneficial effects on productive performance, improved egg quality and diminished the toxic effect of Cd.

**Abstract:**

A total number of 360 laying Japanese quail (8 weeks of age) were randomly divided into 12 groups. Birds in all groups had nearly the same average initial body weight. A factorial arrangement (4 × 3) was performed including four levels of dietary cadmium (Cd) as cadmium chloride (0, 50, 100, and 150 mg/kg diet) and three levels of feed additives (without, 300 mg/kg ascorbic acid and 1.50% natural clay). Results revealed that Cd contaminated feed caused significant (*p* < 0.01) retardation in body weight, lower egg number and egg mass and worse feed conversion. On the other hand, the addition of ascorbic acid or natural clay to quail diets caused a significant (*p* < 0.01) improvement in all studied traits. With respect to the interaction among Cd and the experimental additives, results showed that within each Cd level, ascorbic acid or clay supplementation recorded the highest body weight, egg number, egg weight and mass in addition to improved feed conversion. Cadmium levels decreased (*p* < 0.05) blood total protein, albumen and A/G ratio. Both 300 mg ascorbic acid and 1.50% clay increased (*p* < 0.05) blood total protein and albumen compared to non-supplemented groups. It could be concluded that the consumption of polluted diets Cd causes deleterious effects on the productive performance of laying Japanese quail. The addition of ascorbic acid or natural clay to the diets causes beneficial effects on productive performance traits, improves egg quality criteria and diminishes the toxic effects of Cd.

## 1. Introduction

For commercial use, domestic quails are available in both laying and meat strains [1,2,3]. The reproductive performance of Japanese quail is important in the overall management of the flock [4]. The contamination of the diets and the environment with heavy metals remains a big problem. It affects food safety and subsequently affects the consumers. The contamination of poultry rations with heavy metals causes a high reduction in feed efficiency and egg production, which finally result in a great economic loss for poultry farmers. All potential feed ingredients contain some kinds of heavy metals. Cadmium is considered one of the major environmental pollutants [5]. It is a highly toxic and reactive element which is distributed sparsely in the most of agricultural ecosystems. Once absorbed by humans or animals, Cd is poorly excreted. Great efforts are being made to protect the human food-chain from the entry of cadmium. The excess of Cd intake more than 2 mg/kg diet resulted in elevation in metallothionein synthesis, disturbance of the metabolism of Zn, Ca, and Fe [6]. Cd toxicity induced altered energy, altered behavioral responses, metabolism, kidney damage, anemia, adrenal hypertrophy and cardiac dysfunction [7]. Some reports found that kidney changes occurred independent of bone disease, while others concluded that Cd induced renal dysfunction is the secondary cause of skeletal deterioration [8]. Recently, Saleemi et al. [9] concluded that Cd leads to hepatotoxic and gonadotoxic effects in the quails with dose of 150 and 300 mg/kg feed. As well, researchers found that Cd affects the biochemical parameters of birds. It may decline blood concentrations of total protein and total albumen, A/G ratio and increase the activity of liver enzymes and levels of blood uric acid, urea, and alkaline phosphatase [8,9].

Ascorbic acid is a potent water soluble antioxidant capable of neutralizing and scavenging an array of reactive oxygen species viz., alkoxyl, hydroxyl, superoxide anion, peroxyl, hydroperoxyl radicals, and radicals of reactive nitrogen such as nitroxide, nitrogen di-oxide, and peroxynitrite at very low concentrations [10,11,12]. Ascorbic acid is required for the conversion of vitamin D into its metabolite form (calciterole) which is essential for calcium regulation and the calcification process [13]. It is also is an antioxidant and water-soluble vitamin that is found intra- and extra cellular as ascorbate [14]. In addition, it is a natural antioxidant that prevents the increased production of free radicals induced by oxidative damage to lipids and lipoproteins in various cellular compartments and tissues [15]. Ascorbic acid supplementation at 200 ppm is beneficial for enhancing the immunity, performance, and exploiting the broilers’ full genetic potential [16]. Sharaf et al. [17] concluded that ascorbic acid has potent antioxidant activity against lead and Cd toxicity. They added that the consumption of foods rich in ascorbic acid is a highly recommended to reduce the damage caused by the toxicity with cadmium. 

Supplementing poultry diets with natural clay improves growth performance and egg production rate which is a consequence of the improvement in the nutrient digestibility, feed conversion, ability to make rations more available to the bird and nitrogen retention in bird body, and retarded the absorption of toxic products of digestion that reduce toxicity [18]. Ability of clay to diminish the harmful effects of radiation may have a role in this respect [18]. Abou-Kassem et al. [19] reported that clay supplementation diminished the toxic effects of Cd with levels up to 120 mg/kg of quail diet. The present study aimed to investigate the role of ascorbic acid or natural clay on laying performance, some blood biochemical components and Cd residues in egg components of Japanese quail layers fed diets polluted by Cd at various levels. 

## 2. Materials and Methods

A total number of 360 laying Japanese quail at 8 weeks of age were randomly divided into 12 groups (30 birds/group). Each group was sub-divided into five replicates (6 females each). Each replicate was housed in one cage with area of (2400 cm^2^). A factorial arrangement (4 × 3) was applied including four levels of cadmium chloride (CdCl2); the first group (G0) received zero cadmium level and served as a control group, the other three groups G1, G2, and G3 received 50, 100 and 150 mg/kg diet, respectively). Each group was subdivided into three kinds of feed additives (control, 300 mg ascorbic acid/kg diet and 1.50% natural clay) to study the effect of cadmium, feed additives and their interactions on the productive performance, egg quality and cadmium residues in eggs of laying Japanese quails.

Basal experimental diet was formulated to meet the laying Japanese quail nutrient requirements as recommended by National Research Council (NRC) [20] as shown in Table 1. Vitamin C (Rovimix^®^ Stay-C 35 (Obour city, Egypt); specifically produced for the use as a stabilized source of vitamin C in feed; L ascorbic acid), according to the manufacturers’ guidelines. Cadmium chloride (CdCl2, 2.5 H2O) imported from Chem-Lab NV, Industriezone, De Arend 2, B-8210 Zedelgem, Batch Nr.: 23.5852506, Belgium. Natural clay (from Adwia Company LTD, Obour city, Egypt, Batch No. E09860200) analysis as soluble cations and anions (milligram equivalent (meq)/ 100 gm dry matter soil) were Mg++ 0.25, Ca++ 0.75, K+ 0.10, Na+ 0.05, So4 0.30, Cl 0.55, and HCO3 0.75. Exchangeable cations (meq/100 g dry matter soil) was 2.65 and available nutrients (mg /100 g dry matter soil) were K 1.2, Mn 2.4, P 5.0, Cu 0.30, Zn 0.74, and Fe 0.55 mg [21]. The birds were fed the contaminated diets from 8 to 20 weeks of age. While, at the period from 21 to 24 weeks of age, birds were fed diets without cadmium addition. Birds were reared during the experimental periods under the same management, hygienic and environmental conditions. The bird’s health status was monitored throughout the trial. Quails were exposed to 16 hours of light per day; fed ad-libitum and fresh water was available during the experimental periods. Drinkers and feeding troughs were daily cleaned.

### 2.1. Laying Performance

Egg number and egg weight were daily recorded per replicate in each pen from 8 weeks of age up to the end of the experiment (24 weeks of age). Egg mass (g) was obtained by multiplying egg number by the average egg weight in each pen per day (8–24 weeks of age). Feed conversion during the experimental period (8–24 weeks of age) was calculated for egg production as follows:

Feed conversion for egg produced = g feed/ g egg produced.

Mortality rate of laying experimental periods was calculated as follows:

M.R = N_1_ − N_2_/N_1_ (1)

where, M.R—Mortality rate; N_1_—The number of birds at the beginning of laying period; N_2_—The number of birds at the end of laying period.

### 2.2. Blood Biochemical Components

At 20 and 24 weeks of age, blood samples were collected from sacrificed quails into heparinized sterile tubes. Samples then centrifuged at 3500 rpm for 15 min and serum was separated, collected and stored at −20 °C till examination. Total albumin, protein, urea-N, creatinine, uric acid, aspartate transaminase (AST), alanine transaminase (ALT), and alkaline-phosphatase activities (ALP) were assessed using biodiagnostic commercial kits provided from Biodiagnostic Company (29 El-Tahrir St. Dokki, Giza, Egypt) Batch No: ALT (cat#AL1031), AST (cat#AS1061) according to the manufacturers’ guidelines (REF: 264 001, 264 002) and a spectrophotometer (Shimadzu). The globulin concentrations and A/G ratio were calculated from the difference between the concentrations of total protein and albumin.

### 2.3. Cadmium Residue in Egg Components

At 20 and 24 weeks of age, Cd residues in egg components were determined. Twenty eggs from each treatment group were randomly taken, broken, and egg components were separated (white, yolk and shell) using a separation funnel and directly put in plates then transferred to an oven at 70 °C for 24 h or till a constant dray mass was achieved [22]. The dried samples were perfectly ground and homogenized to be prepared for digestion and residues determination. Cadmium residues were estimated in eggs collected from each treatment group (μg/g wet weight, ppm) in the Central Laboratory of the Faculty of Veterinary Medicine, Zagazig University, Egypt. One gram of each egg component sample (white, yolk, and shell) was placed in a clean screw capped glass bottle and digested with a 4 mL of digesting solution (nitric/per chloric acids, 1:1). Initial digestion was carried out for 24 h at room temperature followed by heating at 110 °C for 2 h. After cooling, deionized water was added, then the solutions were warmed in water bath for 1 h to expel nitrous gases. Digests were then filtered (Whatman No. 1, Ashless, grade 42, cat# 1442-110), and diluted in water to 25 ml deionized water [23]. The obtained solutions were analyzed by flame atomic absorption spectrophotometer (FAAS), (PerkinElmer, 520 South Main St., Suite 2423, Akron, Ohio 44311, USA, Model 2380, Serial No. 131865) to measuring the level of Cd residues in each egg components for all treatment groups. The laboratory had established a calibration programme, which reviewed and adjusted as necessary in order to maintain confidence in the status of calibration, whereas, the laboratory follows international standard Iso/IEC, 17025.

### 2.4. Statistics

Data were statistically analyzed on a 4 × 3 factorial arrangement basis according to Snedecor and Cochran [24] using the following model:

Y_ijk_ = µ + A_i_ + S_j_ + AS_ij_ + e_ijk_ (2)

where Y_ijk_ = an observation, µ = the overall Mean, A_i_ = effect of cadmium level (i = 1 to 4), S_j_ = effect of feed additives (j = 1 to 3), AS_ij_ = the interaction between cadmium level and feed additives (ij = 1 to 12) and e_ijk_ = random error. Differences among means within the same factor were tested using Duncan’s New Multiple Range test [25]. 

## 3. Results

### 3.1. Productive Performance

Significant (*p* < 0.05 and *p* < 0.01) reduction was shown in live body weight with increasing the dietary Cd level at 12, 20, and 24 weeks of age compared to the control (Table 2). Final body weight of laying Japanese quail fed diet supplemented with ascorbic acid or clay was significantly (*p* < 0.05 or 0.01) increased comparing to those fed the control diet (Table 2). 

### 3.2. Mortality Rate:

Mortality rate was significantly (*p* < 0.01) affected by Cd polluted diet during the experimental period of 8–20 weeks of age. The highest value was 12.50% with the high Cd contaminated level (150 mg/kg diet). Results in Table 2 showed that mortality rate was not significantly affected by the addition of ascorbic acid or natural clay to the diet during all experimental periods. Mortality rate was not affected by the interaction among Cd level and ascorbic acid or natural clay in the diet at 20 w.

### 3.3. Egg Number

Egg number was significantly (*p* < 0.05) decreased in quails as the concentration of the Cd increased at all the experimental periods. Ascorbic acid or clay supplementation significantly (*p* < 0.05) increased egg number comparing to hens fed on a diet without supplementation during all the experimental periods (Table 2). The results revealed that egg number was not significantly affected by the interaction between feed additives and Cd pollution during the whole experimental period (Table 3). Within any Cd level, ascorbic acid or clay supplementation increased egg number compared to the other groups. 

### 3.4. Egg Weight

Egg weight was significantly (*p* < 0.05) decreased with increasing Cd level during the experimental period (Table 2). Ascorbic acid or clay supplementation (*p* < 0.01) increased egg weight comparing to the control during the whole period (8–20) and 20–24 weeks of age. Whilst, egg weight values were insignificantly improved by ascorbic acid or clay addition during the other periods (Table 2). Results showed that egg weight was not significantly affected by the interaction between feed additives and Cd contamination during the whole experimental period (Table 3). 

### 3.5. Egg Mass

Quails exposed to Cd had decreased (*p* < 0.01) egg mass as compared to the control throughout the experimental periods and the whole experimental period (Table 4). Results in Table 4 showed that ascorbic acid or clay supplementation significantly (*p* < 0.01) improved egg mass.

While, the interaction between main the two factors insignificantly affected egg mass values. Results indicated that quails fed diet without Cd with 300 mg ascorbic acid/kg diet produced higher egg mass value (8.45 g/day/bird). Hens fed diet contained 150 mg cadmium/kg supplemented with 1.50% clay produced the lowest egg mass value (4.39 gm/day/bird).

### 3.6. Feed Conversion Ratio

With increasing cadmium level in quail diets, feed conversion became significantly worst per egg unit (*p* < 0.05) throughout the experimental periods and the whole period except the insignificant effect during 13–16 weeks of age (Table 4). A significant improvement (*p* < 0.01) was found in feed conversion as a result to ascorbic acid or clay supplementations during the whole experimental period (8–20 weeks of age) and the period of 21–24 weeks of age. However, the diet had 300 mg ascorbic acid recorded the best (*p* < 0.05) feed conversion (3.27 and 3.15 gm feed/gm/egg) values during 8–20 and 21–24 weeks of age, respectively compared to the other experimental groups. 

### 3.7. Blood Biochemical Components

Cadmium levels at 100 and 150 mg/kg diet decreased (*p* < 0.05) blood total protein, total albumen and A/G ratio compared to the control and 50 mg cadmium/kg diet at 20 weeks of age. Meanwhile, cadmium did not affect total globulin. On the other hand, creatinine level decreased (*p* < 0.05) at various cadmium levels compared to the control (Table 5, Table 6, Table 7 and Table 8). Concerning the effect of feed additives at 20 weeks of age, both 300 mg ascorbic acid and 1.50% Clay increased (*p* < 0.05) total protein and albumen compared to the non-supplemented group but did not affect total globulin and A/G ratio level at both 20 and 24 weeks of age. Increasing cadmium levels increased (*p* < 0.05) ALT, AST, ALP, urea-N, and creatinine as compared to the control at 20 and 24 weeks of age. At 20 weeks of age, both 300 mg ascorbic acid and 1.50% Clay decreased (*p* < 0.05) serum ALT, AST and urea-N compared to the non-supplemented group but did not affect ALP and creatinine level at both 20 and 24 weeks of age. The interaction between cadmium level and feed additives had no effect on various serum parameters. 

### 3.8. Cadmium Residues in Egg Components

Pollution of quail diets with Cd had a highly significant (*p* < 0.01) effect on cadmium residues in egg components (white, yolk and shell) at 20 and 24 weeks of age as shown in Table 9. Data showed that ascorbic acid or natural clay had a significant (*p* < 0.01) effect on cadmium residues in egg components. Results showed that Cd residues in egg components at 20 and 24 weeks of age were significantly (*p* < 0.01) affected by the interaction between Cd level and the dietary supplementation of ascorbic acid or natural clay. The highest value recorded (37.59 ± 2.03 mg) was found in yolk with high dietary Cd level (150 ppm) and without additives at 20 weeks of age.

## 4. Discussion

The body weight loss produced by Cd polluted diets (Table 2) might be due to the wide toxic effect of Cd on the whole-body processes in the bird. It has been reported that long term Cd exposure causes depletion of liver and muscular glycogen due to its action on the enzymes involved with glycogenesis and energy metabolism and inducing the oxidative stress in liver and kidney [26]. Also, decreasing body weight might be mediated by the Cd effect on the small intestine, where the nutrients are absorbed or through a dysfunction in the renal tubules resulted from the destructive effect of Cd on kidney. It would lead to an enhanced urinary excretion of some food nutrients which causes a decrease in the utilization of these nutrients in the body [26,27]. Decreasing body weight was also recorded in Japanese quail layers exposed to graded dietary Cd concentration of 40, 80, and 120 ppm [28]. Author attributed this result to the different doses of administration. Some studies on poultry have shown that supplemental ascorbic acid given in feed or by injection improved performance of chickens. Dietary supplementation with ascorbic acid increased the growth rate by about 4.5% [29]. These results agree with Shit et al. [30] who found that dietary L-ascorbic acid supplementation significantly (*p* < 0.05) increased body weight as of laying Japanese quail compared to the control group. On the other hand, these results disagree with those of Attia et al. [31] who reported that the addition of ascorbic acid had no effect on the body weight of broiler chicks until 42 d of age. The improvement in live body weight by clay may be due to delaying the transit time of digesta through the digestive tract by 2 to 2.5 h and promoting absorption of nutrients. 

Digestibility of organic matter, fat, and nitrogen free extract and the nitrogen utilization were increased by supplementary Zeolite (aluminosilicate mineral) [32]. The present results agree with those obtained by Ayyat et al. [33] who found that supplementing clay (1%) in silver Montazah layers diet improved body weight. At any Cd level, ascorbic acid or clay supplementation in quail layer diets recorded higher body weight than the un-supplemented one. The results showed that Cd poisoning can be partially reduced by providing supplementary ascorbic acid or natural clay. 

According to our results, Cd polluted diets caused 12.50% mortality rate. This result is in disagreement with Bokori et al. [7] who stated that dietary Cd pollution of 25 or 75 ppm did not cause indicative signs of mortality rate in chickens as compared to the control during the experimental period for nine months ago. Due to Klassen and Liu [34], the lethality associated with exposure to cadmium is related to cardiotoxicity and hepatotoxicity. Contrarily, Erdogan et al. [15] clarified that mortalities were not related to toxicity of Cd because no abnormal signs were observed during clinical observation or at autopsy examination in broiler chicks. Ascorbic acid has been reported to protect cells involved in the immune response such as lymphocytes, macrophages and plasma cells against oxidative damage of quail layer cells. Some studies on poultry have shown that supplemental ascorbic acid given in feed or drinking water or by injection of chickens reduced the mortality percentage by about 5% [29].

The significant decrease in egg number in the Cd polluted diets Japanese quail found in the present study (Table 2) could have resulted from alterations in the egg formation pathway or may be through the suppression of calcium metabolism [35]. Similarly, Rahman et al. [35] showed reduced egg production in quails. Decreased egg production was also observed in laying hens with single dietary exposures of 60 ppm radioactive Cd [36]. The dose and period of Cd exposure may be an important factor altering egg production potentialities which could be confirmed by the findings that showed no effect of Cd contamination on the egg production of pheasants given drinking water containing 1.5 mg cadmium/L for only 12 weeks [37]. Ascorbic acid or clay supplementation significantly increased egg number (Table 2). These results agree with Njoku and Nwazota [38] who clarified that ascorbic acid supplementation to laying hens’ diets caused a proportional increase in the number of eggs laid. The presence of antioxidant (ascorbic acid) could adversely inhibit the oxidative protein denaturation and improve nutrients digestibility and feed efficiency. It could be also a reason for rising layers performance [39]. Present results agree with Moghaddam et al. [40] who found that dietary Zeolite addition at levels of 1.5%, 3%, and 4.5% caused a significant (*p* < 0.01) increase in egg production of Hy-Line hens. Therefore, supplementation of Zeolite can be used to extract ammonia by ion exchange which helps to remove stress-forming ammonia from the hen intestine and thereby increasing egg production [41]. On the other hand, Lemser et al. [42] indicated that supplementation of rye-based broiler diets with natural clay minerals showed a negative effect on egg production. 

Generally, the results indicated that the use of the cation exchange is capable to reduce the uptake and influence the distribution of heavy metals in poultry tissues. Evans et al. [43] concluded that the use of synthetic and natural Zeolite improved the performance of poultry. In line with our results, Rahman et al. [35] showed that egg weight of Japanese quail layers was markedly affected by Cd administration with injection levels of 0.1, 0.3, 1, 3, and 10 mg/kg body weight/day up to the end of the experimental period (14 days). Authors attributed this action to alterations in the egg formation pathway. This is may also returned to that quails exposed to Cd pollution produced fewer eggs and had lower egg weights than controls. Ascorbic acid or clay supplementation significantly (*p* < 0.01) increased egg weights (Table 2). This is because quails fed diets supplemented with ascorbic acid or clay produced more eggs and had higher egg weights than those fed diet without feed additives. These results are confirmed by Njoku and Nwazota [38] who clarified that ascorbic acid supplementation to laying hens’ diets (250 ppm) showed highest mean egg weight but statistically no variation was drawn among the groups. According to Elliot and Edwards [44], natural Zeolite as a Clinoptolite bearing rock material was found to increase egg weight by incorporation in the hens’ diet at an inclusion rate of less than 10%. Moghaddam et al. [40] found that dietary added Zeolite in levels of 1.5, 3 and 4.5% caused a significant (*p* < 0.05) increase in egg weight of Hy-Line hens. In contrast, Ozturk et al. [45] found that dietary Zeolite (aluminosilicate mineral) supplementation of 0%, 2%, 4%, 6%, and 8% as Clinoptilolite had no significant effect on egg weight of Babcock laying hens. The obtained results reported decreased egg mass due to Cd pollution (Table 4). It is worth noting that egg mass significantly decreased (*p* < 0.01) with increasing dietary Cd level which agree with results reported by Baykov et al. [46]. In a converse trend, results showed increased egg mass due to ascorbic acid or clay supplementation. These results agree with Moghaddam et al. [41] who demonstrated that Zeolite at levels of 1.5, 3 and 4.5% caused significant (*p* < 0.01) increases in egg mass of Hy-Line hens. 

The poor feed conversion due to Cd pollution could be due to the highly significant decrease in egg mass as exposed to Cd pollution. Long-term cadmium exposure causes depletion of liver and muscular glycogen. This effect might reduce nutrient metabolism and feed utilization [35]. Feed conversion value depends mainly on the amount of feed consumed and the egg mass. In this context, it is worth noting that egg mass in ascorbic acid or clay groups were significantly (*p* < 0.05) higher than un-supplemented group (Table 4). These results are in harmony with Denli et al. [47] who found that dietary ascorbic acid improved feed conversion ratio in laying hens under stress. On the contrary, Soltani et al. [48] postulated that values of feed conversion rate of laying hens were not affected by dietary ascorbic acid (*p* > 0.05) with level of 250 mg/kg as compared to the control group. 

For blood parameters, our results in Table 7 and Table 8 fully agree with those obtained by Abou-Kassem et al. [19] who found that the increased levels of cadmium in quail diets significantly decreased (*p* < 0.05) total protein, total albumen and A/G ratio compared to the control group with no effects of various levels of clay or vitamin E supplementations. Also, Hashem et al. [49] found that cadmium levels at 100 mg/kg diet statistically decreased serum total proteins, albumin and globulins values. It was clear that cadmium levels at 100 and 150 mg/kg diet significantly increased (*p* < 0.05) ALT, AST, uric acid, urea-N, creatinine, and alkaline phosphatase levels compared to control and 50 mg cadmium/kg diet at 20 and 24 weeks of age of Japanese quails. Urea-N appears to be the most useful variable for the detection of pre-renal causes of renal failure with Cd toxicity. But the effects of feed additive and the interaction between cadmium level and feed additive at 20 and 24 weeks of age were not affecting (*p* > 0.05) on these traits (Table 5 and Table 6). In a partially disagreement, Rambeck and Kollmer [50] showed that the addition of dietary ascorbic acid had a great protective effect on kidney damage from cadmium intake. Hashem et al. [49] reported that group received cadmium at level of 100 mg /kg diet significantly increased (*p* < 0.05) blood ALT, AST, uric acid and creatinine level compared to control group. The increase in ALT and AST enzyme levels and the outflow of these enzymes to the blood from the liver due to Cd hepatotoxic effect is considered as an indicator of hepatocellular damage. ALT activity was significantly increased, while ALP activity was significantly decreased in plasma of rats fed 5 mg Cd /kg [51]. Cadmium revealed a significant increase in ALP activities when compared to the control group (Table 6). Our results assure the prophylactic potential of ascorbic acid or clay to prevent or decrease cadmium-induced toxic manifestations. ALP in blood is considered as an indicator of mineral status and bone mineralization especially Ca and P which plays an important role in the homeostasis of the body and ensures appropriate conditions for biological activities such as energy utilization, nucleic acid synthesis and bone mineralization [52].

The hepatocellular injury due to cadmium could be attributed to the cadmium-induced generation of free radicals [53]. The activity of AST and ALT enzymes used as stress indicators of evaluation for impairment and damage of tissue liver which produced from Cd toxicity and attributed the elevated activities of ALT and AST enzymes to the outflow from the liver cytosol to the blood. Also, increasing AST and ALT attributed to the hepatotoxic effect, hepatocellular damage or cellular degradation by Cd in quail liver. Toxicity with cadmium may be due to changes in liver carbohydrate metabolism especially activation of liver glycogenolysis and glycolysis as well as increased levels of plasma glucose [54]. From these explanations, the linkage between liver damage, energy metabolism, productive traits, and the residues of cadmium in quail egg components is clarified. Conversely, Erdogan et al. [15] reported that different Cd levels had no effects on AST and ALT activities. 

Long-term exposure to cadmium leads to pathological changes in the quail kidneys which contains morphological changes are initially limited to proximal tubular epithelial cell degeneration followed by cellular atrophy, interstitial fibrosis, and glomerular sclerosis and finally associated with biochemical evidence of renal tubular dysfunction. These explanations ensured by Abdo and Abdalla [55] and Abou-Kassem et al. [19] who reported that kidney function tests elevated in Cd treated groups indicating the toxic effect of cadmium which was presumably due to nephrotoxic effect of cadmium on renal tubules and glomeruli. 

Sato et al. [56] postulated that Cd transfer from laying hen to eggs was restricted after the maternal bird was exposed to Cd. furthermore; Cd accumulates in the follicle walls of ovary. These results suggest that the follicle walls might play a role in protecting the follicle yolks against Cd toxicity. These findings agree with Herzig et al. [57] who found that Cd level of all investigated tissues (liver and leg muscle) were significantly (*p* < 0.05) increased when chicken received 1.47 mg Cd within 10 days. Results of this study are in disagreement with Toman et al. [37] who found that the hepatic Cd concentration of pheasants exposed to Cd (1.5 mg/ L water) were under the limit of detection (0.01 mg/kg) in all sample collections (after 4, 8, and 12 w of the experiment). 

Our results showed that ascorbic acid or natural clay had a significant (*p* < 0.01) effect on cadmium residues in egg components. On the contrary, Erdogan et al. [15] showed that ascorbic acid supplementation to the diet did not prevent Cd accumulation in several organs of chicks. These results agree with Attia et al. [31] who reported that natural clay addition to lead contaminated diets clearly reduced the level of lead residues in the viscera and muscles. Natural clay prevents the lead toxicity by reducing lead absorption in the intestinal tract and increasing fecal excretion [31]. 

## 5. Conclusions

It can be concluded that the consumption of polluted diets with heavy metals such as Cd causes deleterious effects on the productive performance of laying Japanese quails. The addition of ascorbic acid or natural clay to the diet of laying Japanese quails caused beneficial effects on productive performance and diminished the toxic effect of Cd on productive results during the treatment period. 

Compliance with Ethical Standards: All procedures by this study were in accordance with international ethical standards. The research involved no human participants.

## Figures and Tables

**Table 1:** Composition and chemical analysis of experimental diet (8–24 w).

Ingredients	Percentages
Yellow corn	56.50
Soy bean meal (44%)	30.10
Corn gluten (60%)	3.00
Cotton seed oil	2.50
Limestone	5.00
Dicalcium phosphate	2.30
Nacl	0.30
Premix *	0.30
Calculated analysis **	
C.P%	20.01
ME. Kcal/kg	2894.00
Ca%	2.51
P% Avail, P.	0.54
Lysine	1.04
Met. + Cys. %	0.69

* Layer Vitamin and Mineral premix Each 3 kg consists of: Vit A. 12 Mi.U, Vit E. 15 IU., Vit. D_3_ 4 Mi.U, Vit, B_1_ 1g, Vit, B_2_ 8g, Pantothenic acid 10.87g, Nicotinic acid 30g, Vit. B_6_ 2g, Vit. B_12_ 10 mg. Folic acid 1 g, Biotin 150 mg, Copper 5g, Iron 5g, Manganese 70 g Iodine 0.5g, Selenium 0.15 g, Zinc 60 g. Antioxidant 10 g; ** Calculated according to NRC [20].

**Table 2:** Live body weight changes, egg number, egg weight and mortality rate of Japanese quail layers as affected by dietary cadmium levels, some feed additives during the experiment (8–24 weeks of age).

Items	Body Weight (g)					M.R	Egg Number					Egg Weight (g)				
	8 w	12 w	16 w	20 w	24 w	8–20 w	8–12 w	13–16 w	17–20 w	8–20 w	21–24 w	8–12 w	13–16 w	17–20 w	8–20 w	21–24 w
Cadmium level (mg/kg diet)																
Control	194.78	192.66 ^a^	190.84	197.12 ^b^	207.16 ^ab^	0.00	16.83 ^b^	17.33 ^b^	18.17 ^b^	17.45 ^b^	17.92 ^b^	10.61 ^a^	10.72 ^a^	10.80 ^b^	10.71 ^a^	10.90 ^a^
50 mg Cd/kg	194.96	190.20 ^b^	190.67	196.21 ^b^	205.89 ^b^	0.00	17.25 a	17.06 ^bc^	17.69 ^bc^	17.33 ^b^	17.42 ^c^	10.56 ^a^	10.86 ^a^	10.99 ^a^	10.81 ^a^	11.00 ^a^
100 mg Cd/kg	194.68	187.57 ^c^	189.01	196.28 ^b^	205.54 ^b^	4.16 ^b^	16.47 ^b^	16.72 ^c^	17.06 ^c^	16.75 ^c^	17.22 ^cd^	10.43 ^b^	10.67 ^a^	10.72 ^b^	10.60 ^b^	10.86 ^a^
150 mg Cd/kg	194.53	185.03 ^d^	188.01	195.15 ^b^	203.89 ^c^	12.50 ^a^	15.42 ^c^	15.50 ^d^	15.33 ^d^	15.42 ^d^	15.69 ^d^	10.31 ^c^	10.36 ^b^	10.29 ^c^	10.32 ^c^	10.51 ^b^
SEM	0.81	1.56	1.91	1.48	1.89	0.18	1.09	0.55	0.48	0.37	0.54	0.18	0.21	0.32	0.13	0.29
*p*-value	0.814	0.024	0.568	0.370	0.019	0.001	0.001	0.004	0.007	0.001	0.002	0.001	0.008	0.001	0.006	0.018
Feed additives																
Control	194.13	187.47	188.48	194.20	203.83 ^b^	0.00	15.96 ^b^	15.79 ^c^	15.90 ^c^	15.88 ^c^	16.48 ^c^	10.44	10.61	10.62	10.55	10.72
300 mg.vit C	195.20	190.15	191.19	198.64	207.85 ^ab^	0.00	17.37 ^a^	17.73 ^a^	18.50 ^a^	17.86 ^a^	18.73 ^a^	10.55	10.78	10.84	10.72	10.94
1.50% clay	195.07	189.63	190.71	197.45	208.97 ^a^	0.00	17.28 ^a^	17.35 ^b^	17.90 ^b^	17.47 ^b^	17.67 ^b^	10.51	10.66	10.73	10.63	10.94
SEM	0.63	1.69	1.74	1.17	1.60	0.00	0.35	0.50	0.44	0.52	0.73	0.31	0.24	0.28	0.20	0.34
*p*-value	0.231	0.367	0.501	0.013	0.009	0.918	0.001	0.005	0.011	0.001	0.001	0.547	0.417	0.842	0.028	0.067

^a–d^ Values followed by different letters in each column within main effect are significantly different (*p* < 0.05); M.R: mortality rate.

**Table 3:** Live body weight, egg number, and egg weight of Japanese quail layers as affected by interaction between dietary cadmium levels and some feed additives during the experiment (8–24 weeks of age).

Items	Body Weight g/bird					M.R	Egg Number/Bird					Egg Weight (g)				
	8 w	12 w	16 w	20 w	24 w	8–20 w	8–12 w	12–16 w	16–20 w	8–20 w	21–24 w	8–12 w	12–16 w	16–20 w	8–20 w	21–24 w
Interaction effect																
Without cadmium																
Control	194.78	192.66	190.84	197.12	207.16	0.00	16.83	17.33	18.17	17.45	17.92	10.61	10.72	10.80	10.71	10.90
300 mg.vit C	195.20	194.42	194.13	201.09	213.75	0.00	19.08	19.67	20.83	19.86	20.92	10.78	10.95	11.07	10.93	11.20
1.50% clay	193.01	193.50	193.43	200.02	210.32	0.00	18.67	18.67	19.83	19.05	19.08	10.65	10.83	10.89	10.79	11.07
50 mg Cadmium																
Control	192.34	188.47	189.67	195.14	202.46	4.17 ^b^	15.83	15.42	15.67	15.64	17.17	10.49	10.89	10.95	10.78	10.96
300 mg.vit C	194.98	190.70	190.33	197.19	205.33	0.00	18.08	17.92	18.58	18.20	18.83	10.58	10.88	11.03	10.83	11.01
1.50% clay	196.97	191.44	192.01	196.30	206.30	0.00	17.83	17.83	18.83	18.17	19.25	10.61	10.82	11.00	10.81	11.04
100 mg Cadmium																
Control	192.71	185.23	187.45	193.21	198.20	8.34 ^a^	16.00	15.50	15.66	15.56	15.58	10.37	10.53	10.51	10.47	10.64
300 mg.vit C	193.37	189.17	191.58	198.71	206.20	4.17 ^b^	16.92	17.83	18.58	17.78	18.83	10.47	10.85	10.91	10.74	10.95
1.50% clay	191.97	188.30	188.01	196.91	209.23	4.17 ^b^	16.50	16.83	17.41	16.92	17.25	10.44	10.63	10.74	10.60	10.99
150 mg Cadmium																
Control	192.70	183.51	185.94	191.34	195.52	8.34 ^a^	15.17	14.92	14.58	14.89	15.25	10.28	10.29	10.21	10.26	10.39
300 mg.vit C	193.25	186.29	188.70	197.57	204.11	4.17 ^b^	15.42	15.50	15.92	15.61	16.33	10.35	10.43	10.36	10.38	10.61
1.50% clay	194.73	185.30	189.40	196.56	206.04	4.16 ^b^	15.67	16.08	15.50	15.75	15.50	10.30	10.36	10.28	10.31	10.53
SEM	0.97	3.21	4.07	2.38	2.71	0.17	0.93	0.71	1.03	0.67	1.08	0.27	0.24	0.30	0.19	0.21
*p*-value	0.910	0.715	0.640	0.553	0.418	0.324	0.298	0.354	0.209	0.117	0.870	0.227	0.324	0.108	0.487	0.144

^a,b^ Values followed by different letters within each effect in each column are significantly different (*p* < 0.05); M.R: mortality rate.

**Table 4:** Egg mass and feed conversion of Japanese quail layers as affected by dietary cadmium levels, some feed additives during the experiment (8–24 weeks of age).

Items	Egg Mass g/day/bird					Feed Conversion g feed/g/egg				
	8–12 w	12–16 w	16–20 w	8–20 w	21–24 w	8–12 w	13–16 w	17–20 w	8–20 w	21–24 w
Cadmium level										
Control	6.38 ^a^	6.63 ^a^	7.00 ^a^	6.67 ^b^	6.98 ^b^	3.25 ^c^	3.26	3.29 ^c^	3.27 ^d^	3.20 ^c^
50 mg Cd/kg	6.51 ^a^	6.61 ^a^	6.94 ^a^	6.69 ^b^	7.24 ^a^	3.30 ^c^	3.27	3.24 ^c^	3.27 ^d^	3.23 ^c^
100 mg Cd/kg	6.13 ^b^	6.38 ^b^	6.54 ^b^	6.35 ^c^	6.64 ^c^	3.46 ^b^	3.41	3.41 ^b^	3.42 ^b^	3.29 ^b^
150 mg Cd/kg	5.68 ^c^	5.74 ^c^	5.63 ^b^	5.68 ^d^	5.44 ^d^	3.65 ^a^	3.51	3.53 ^a^	3.57 ^a^	3.45 ^a^
Feed additives										
Control	5.95 ^b^	5.98 ^b^	6.04 ^b^	5.99 ^b^	6.28 ^b^	3.45	3.34	3.45	3.44 ^a^	3.38 ^a^
300 mg.vit C	6.55 ^a^	6.83 ^a^	7.17 ^a^	6.85 ^a^	7.33 ^a^	3.26	3.37	3.23	3.27 ^c^	3.15 ^c^
1.50% clay	6.44 ^a^	6.61 ^a^	6.87 ^ab^	6.64 ^b^	6.60 ^b^	3.39	3.32	3.31	3.34 ^b^	3.29 ^b^
Interaction effect										
Without cadmium										
Control	6.38	6.63	7.00	6.67	6.98 ^c^	3.25	3.26	3.29	3.27	3.20
300 mg ascorbic acid	7.35	7.69	8.24	7.76	8.45 ^a^	2.77	3.43	2.96	3.06	2.99
1.50% Clay	7.09	7.22	7.71	7.34	7.55 ^b^	3.16	3.20	3.18	3.18	3.32
50 mg Cadmium										
Control	5.94	6.00	6.13	6.02	6.72 ^c^	3.31	3.31	3.33	3.32	3.31
300 mg ascorbic acid	6.84	6.95	7.31	7.03	7.41 ^b^	3.28	3.25	3.16	3.23	3.13
1.50% Clay	6.76	6.89	7.38	7.01	7.60 ^b^	3.30	3.25	3.23	3.26	3.24
100 mg Cadmium										
Control	5.92	5.83	5.69	5.81	5.79 ^b^	3.52	3.55	3.57	3.54	3.43
300 mg ascorbic acid	6.32	6.90	7.24	6.82	7.36 ^b^	3.41	3.30	3.30	3.34	3.23
1.50% Clay	6.15	6.40	6.68	6.41	6.77 ^c^	3.46	3.38	3.34	3.39	3.21
150 mg Cadmium										
Control	5.57	5.48	5.32	5.46	5.66 ^d^	3.72	3.60	3.62	3.64	3.57
300 mg ascorbic acid	5.70	5.77	5.89	4.79	6.18 ^d^	3.60	3.50	3.48	3.53	3.39
1.50% Clay	5.77	5.95	5.69	5.81	4.39 ^e^	3.64	3.44	3.48	3.52	3.38
SEM	0.34	0.39	0.43	0.28	0.51	0.19	0.22	0.29	0.15	0.13
*p*-value										
Cadmium level	0.006	0.001	0.001	0.002	0.021	0.041	0.137	0.007	0.009	0.029
Feed additives	0.003	0.001	0.001	0.002	0.034	0.119	0.225	0.362	0.048	0.043
Interaction effect	0.320	0.524	0.182	0.417	0.019	0.294	0.381	0.615	0.540	0.343

^a–d^ Values followed by different letters within each effect in each column are significantly different (*p* < 0.05).

**Table 5:** Some blood parameters of Japanese quail layers as affected by dietary cadmium levels, feed additives and their interactions at 20 weeks of age.

Items	ALT, U/L	AST, U/L	Uric Acid, Mg/dL	Urea-N, Mg/dL	Creatinine Mg/dL	Alkaline Phosphatase U/L
Cadmium level (mg/kg diet)						
Control	33.88 ^c^	54.83 ^c^	5.42 ^c^	12.95 ^c^	0.70 ^b^	174.55 ^c^
50 mg Cd/kg	34.22 ^c^	54.31 ^c^	5.32 ^c^	13.72 ^c^	0.79 ^a^	176.12 ^c^
100 mg Cd/kg	37.42 ^b^	59.21 ^b^	5.98 ^b^	16.23 ^b^	0.83 ^a^	182.37 ^b^
150 mg Cd/kg	41.95 ^a^	61.90 ^a^	6.63 ^a^	20.47 ^a^	0.87 ^a^	199.11 ^a^
Feed additives						
Control	37.64 ^a^	58.56 ^a^	5.99	16.97 ^a^	0.82	185.16
300 mg.vit C	35.82 ^b^	56.63 ^b^	5.61	14.84 ^b^	0.77	180.60
1.50% clay	36.41 ^b^	56.25 ^b^	5.68	14.84 ^b^	0.79	180.01
Interaction						
Without cadmium						
Control	33.88	54.83	5.42	12.95	0.68	174.55
300 mg ascorbic acid	31.86	52.29	4.89	11.22	0.69	168.10
1.50% Clay	32.94	52.40	5.05	11.18	0.73	167.68
50 mg Cadmium						
Control	35.48	56.56	5.29	13.95	0.82	179.50
300 mg ascorbic acid	35.79	53.22	5.39	13.43	0.77	173.90
1.50% Clay	33.40	53.15	5.27	13.79	0.79	174.95
100 mg Cadmium						
Control	38.50	60.11	6.15	19.26	0.87	185.46
300 mg ascorbic acid	35.95	59.37	5.86	14.92	0.81	182.10
1.50% Clay	37.82	58.16	5.93	14.51	0.79	179.54
150 mg Cadmium						
Control	42.71	62.75	7.10	21.73	0.92	201.11
300 mg ascorbic acid	41.68	61.66	6.32	19.80	0.83	198.32
1.50% Clay	41.46	61.29	6.47	19.89	0.85	197.89
SEM	2.84	2.31	1.10	2.49	0.07	9.11
*p*-value						
Cadmium level	0.001	0.004	0.009	0.001	0.007	0.001
Feed additives	0.023	0.241	0.170	0.047	0.110	0.07
Interaction	0.082	0.173	0.354	0.477	0.209	0.119

^a–c^ Values followed by different letters within each effect in each column are significantly different (*p* < 0.05).

**Table 6:** Some blood parameters of Japanese quail layers as affected by dietary cadmium levels, feed additives and their interactions at the end of (experiment 24 weeks of age).

Items	ALT, U/L	AST, U/L	Uric Acid, Mg/dL	Urea-N, Mg/dL	Creatinine Mg/dL	Alkaline Phosphatase U/L
Cadmium level (mg/kg diet)						
Control	34.65 ^b^	55.18 ^c^	5.43 ^b^	13.03 ^c^	0.71 ^b^	174.92 ^c^
50 mg Cd/kg	32.58 ^c^	54.20 ^c^	5.32 ^c^	12.86 ^c^	0.78 ^b^	174.27 ^c^
100 mg Cd/kg	34.90 ^b^	57.75 ^b^	5.46 ^b^	14.66 ^b^	0.76 ^b^	178.57 ^b^
150 mg Cd/kg	39.92 ^a^	60.76 ^a^	6.34 ^a^	19.58 ^a^	0.83 ^a^	195.36 ^a^
Feed additives						
Control	36.01 ^a^	57.58 ^a^	5.93 ^a^	15.68 ^a^	0.80	183.12 ^a^
300 mg.vit C	34.97 ^b^	56.39 ^b^	5.44 ^b^	14.56 ^b^	0.74	179.11 ^b^
1.50% clay	35.30 ^b^	55.68 ^b^	5.47 ^b^	14.04 ^b^	0.77	177.72 ^b^
Interaction						
Without cadmium						
Control	34.65	55.18	5.43	13.03	0.71	174.92
300 mg ascorbic acid	34.23	52.54	5.39	11.64	0.70	172.15
1.50% Clay	34.01	52.79	5.18	11.13	0.75	168.15
50 mg Cadmium						
Control	33.11	55.36	5.68	12.68	0.80	175.34
300 mg ascorbic acid	32.41	54.16	5.09	12.74	0.79	173.97
1.50% Clay	32.24	53.07	5.20	13.17	0.78	173.51
100 mg Cadmium						
Control	35.25	58.50	5.71	16.14	0.83	183.61
300 mg ascorbic acid	33.82	58.12	5.33	14.66	0.71	176.72
1.50% Clay	35.62	56.64	5.33	13.19	0.75	175.38
150 mg Cadmium						
Control	41.04	61.31	6.89	20.87	0.88	198.63
300 mg ascorbic acid	39.41	60.74	5.96	19.22	0.78	193.61
1.50% Clay	39.32	60.24	6.16	18.67	0.81	193.84
SEM	3.71	3.27	0.93	2.08	0.07	8.75
*p*-value						
Cadmium level	0.001	0.001	0.007	0.001	0.042	0.001
Feed additives	0.015	0.049	0.038	0.020	0.715	0.039
Interaction	0.228	0.109	0.512	0.099	0.617	0.176

^a–c^ Values followed by different letters within each effect in each column are significantly different (*p* < 0.05).

**Table 7:** Some blood parameters as affected by dietary cadmium levels, feed additives and their interactions at 20 weeks of age.

Items	Total Protein, G/dl	Total Albumin, G/dl	Total Globulin, G/dl	A/G ratio
Cadmium level				
Control	5.41 ^a^	3.20 ^a^	2.21	1.46 ^a^
50 mg Cd/kg	5.28 ^a^	3.06 ^a^	2.22	1.39 ^a^
100 mg Cd/kg	5.08 ^b^	2.86 ^b^	2.22	1.30 ^b^
150 mg Cd/kg	4.58 ^c^	2.37 ^c^	2.21	1.08 ^c^
Feed additives				
Control	4.81 ^b^	2.63 ^c^	2.18	1.22
300 mg.vit C	5.32 ^a^	3.11 ^a^	2.21	1.42
1.50% clay	5.12 ^a^	2.87 ^b^	2.87	1.28
Interaction				
Without cadmium				
Control	5.19	2.99	2.20	1.37
300 mg ascorbic acid	5.65	3.54	2.11	1.69
1.50% Clay	5.39	3.05	2.34	1.31
50 mg Cadmium				
Control	5.07	3.05	2.02	1.53
300 mg ascorbic acid	5.55	3.23	2.32	1.41
1.50% Clay	5.22	2.90	2.33	1.24
100 mg Cadmium				
Control	4.72	2.39	2.33	1.02
300 mg ascorbic acid	5.23	3.04	2.19	1.39
1.50% Clay	5.28	3.15	2.13	1.48
150 mg Cadmium				
Control	4.27	2.09	2.18	0.97
300 mg ascorbic acid	4.86	2.63	2.23	1.18
1.50% Clay	4.60	2.38	2.21	1.08
SEM	0.45	0.39	0.15	0.22
*p*-value				
Cadmium level	0.001	0.005	0.211	0.001
Feed additives	0.001	0.006	0.167	0.092
Interaction	0.210	0.090	0.223	0.170

^a–c^ Values followed by different letters in each column are significantly different (*p* < 0.01).

**Table 8:** Some blood parameters as affected by dietary cadmium levels, feed additives, and their interaction at 24 weeks of age.

Items	Total Protein G/dL	Total Albumin G/dL	Total Globulin G/dL	A/G Ratio
Cadmium level				
Control	6.09 ^a^	3.52 ^a^	2.56 ^a^	1.38 ^c^
50 mg Cd/kg	5.98 ^a^	3.60 ^a^	2.37 ^b^	1.54 ^b^
100 mg Cd/kg	5.89 ^b^	3.48 ^b^	2.11 ^d^	1.66 ^a^
150 mg Cd/kg	4.73 ^c^	2.68 ^c^	2.05 ^d^	1.30 ^d^
Feed additives				
Control	5.43 ^c^	3.22	2.21 ^b^	1.47 ^b^
300 mg.vit C	5.81 ^a^	3.41	2.39 ^a^	1.44 ^b^
1.50% clay	5.68 ^b^	3.46	2.22 ^b^	1.57 ^a^
Interaction				
Without cadmium				
Control	6.09	3.52	2.56	1.38
300 mg ascorbic acid	6.60	3.91	2.69	1.46
1.50% Clay	6.11	3.70	2.41	1.56
50 mg Cadmium				
Control	5.79	3.59	2.20	1.68
300 mg ascorbic acid	6.24	3.74	2.51	1.50
1.50% Clay	5.90	3.48	2.41	1.44
100 mg Cadmium				
Control	5.28	3.17	2.11	1.50
300 mg ascorbic acid	5.60	3.48	2.12	1.64
1.50% Clay	5.89	3.80	2.09	1.84
150 mg Cadmium				
Control	4.56	2.59	1.96	1.32
300 mg ascorbic acid	4.80	2.57	2.23	1.15
1.50% Clay	4.83	2.86	1.97	1.45
SEM	0.67	0.81	0.21	0.33
*p*-value				
Cadmium level	0.001	0.001	0.006	0.034
Feed additives	0.005	0.208	0.017	0.009
Interaction	0.254	0.310	0.278	0.189

^a–d^ Values followed by different letters within each effect in each column are significantly different (*p* < 0.05).

**Table 9:** Cadmium residual in tissues of Japanese quail layers as affected by dietary cadmium levels, feed additives and their interaction at 20 and 24 weeks of age.

Items	Cadmium Residual (mg/kg DM)					
	20 w			24 w		
	White	Yolk	Shell	White	Yolk	Shell
Cadmium level (mg/kg diet)						
Control	0.13 ^d^	0.36 ^d^	0.18 ^d^	0.13 ^c^	0.25 ^d^	0.15 ^c^
50 mg Cd/kg	6.41 ^c^	12.43 ^c^	9.57 ^c^	6.21 ^b^	5.32 ^c^	3.52 ^b^
100 mg Cd/kg	14.31 ^b^	17.31 ^b^	16.71 ^b^	7.72 ^b^	11.03 ^b^	7.98 ^b^
150 mg Cd/kg	26.73 ^a^	37.15 ^a^	33.65 ^a^	22.09 ^a^	23.74 ^a^	25.13 ^a^
Feed additives						
Control	14.11 ^a^	19.29 ^a^	17.12 ^a^	10.63 ^a^	12.03 ^a^	10.92 ^a^
300 mg vit C	10.05 ^b^	16.61 ^b^	12.87 ^b^	7.19 ^b^	9.45 ^b^	8.22 ^b^
1.50% clay	9.35 ^c^	15.15 ^c^	13.68 ^c^	6.52 ^b^	8.56 ^c^	6.98 ^c^
Interaction						
Without cadmium						
Control	0.13 ^a^	0.36 ^a^	0.18 ^a^	0.13 ^a^	0.25 ^a^	0.15 ^a^
300 mg ascorbic acid	0.24 ^a^	0.32 ^a^	0.29 ^a^	0.24 ^a^	0.32 ^a^	0.21 ^a^
1.50% Clay	0.21 ^a^	0.28 ^a^	0.25 ^a^	0.22 ^a^	0.21 ^a^	0.22 ^a^
50 mg Cadmium						
Control	8.08 ^bc^	11.74 ^c^	9.43 ^bc^	5.15 ^c^	5.21 ^bc^	3.49 ^bc^
300 mg ascorbic acid	5.70 ^ab^	9.35 ^b^	7.82 ^b^	3.44 ^b^	3.46 ^b^	3.14 ^b^
1.50% Clay	6.68 ^b^	10.61 ^bc^	8.89 ^bc^	3.95 ^bc^	5.76 ^c^	3.17 ^b^
100 mg Cadmium						
Control	16.31 ^d^	23.20 ^e^	20.09 ^d^	13.17 ^d^	16.24 ^de^	13.61 ^d^
300 mg ascorbic acid	9.92 ^c^	14.88 ^d^	12.70 ^cd^	6.46 ^cd^	9.93 ^d^	5.26 ^cd^
1.50% Clay	9.19 ^c^	14.34 ^d^	11.62 ^c^	4.29 ^bc^	6.47 ^cd^	4.51 ^c^
150 mg Cadmium						
Control	29.43 ^f^	37.99 ^g^	34.94 ^f^	23.08 ^g^	25.89 ^g^	28.17 ^g^
300 mg ascorbic acid	25.06 ^de^	33.75 ^fg^	30.83 ^e^	20.72 ^f^	23.80 ^f^	24.03 ^f^
1.50% Clay	23.07 ^de^	31.64 ^f^	30.74 ^e^	19.03 ^e^	21.02 ^e^	23.11 ^e^
SEM	1.86	2.94	2.33	2.59	1.40	1.66
*p*-value						
Cadmium level	0.001	0.001	0.001	0.003	0.001	0.002
Feed additives	0.008	0.001	0.001	0.011	0.01	0.008
Interaction	0.001	0.001	0.001	0.011	0.008	0.001

^a–g^ Values followed by different letters within each effect in each column are significantly different (*p* < 0.05).
